# Low‐Dose, Contrast‐Enhanced Mammography Compared to Contrast‐Enhanced Breast MRI: A Feasibility Study

**DOI:** 10.1002/jmri.27079

**Published:** 2020-02-14

**Authors:** Paola Clauser, Pascal A.T. Baltzer, Panagiotis Kapetas, Mathias Hoernig, Michael Weber, Federica Leone, Maria Bernathova, Thomas H. Helbich

**Affiliations:** ^1^ Department of Biomedical Imaging and Image‐Guided Therapy Medical University of Vienna Vienna Austria; ^2^ Diagnostic Imaging, Siemens Healthcare GmbH Forchheim Germany; ^3^ Department of Biomedical Imaging and Image‐Guided Therapy, Division of General and Pediatric Radiology Medical University of Vienna Vienna Austria; ^4^ ASST Fatebenefratelli‐Sacco Ospedale Fatebenefrateli e Oftalmico Milan Italy

**Keywords:** contrast‐enhanced mammography, contrast‐enhanced MRI, breast cancer, radiation dose

## Abstract

Contrast‐enhanced MRI (CE‐MRI) is the most sensitive technique for breast cancer detection. Contrast‐enhanced mammography (CEM) is emerging as a possible alternative to CE‐MRI.

**Purpose:**

To evaluate the diagnostic performance of a low radiation dose contrast‐enhanced mammography (L‐CEM) in women with suspicious findings on conventional imaging compared to CE‐MRI of the breast.

**Study Type:**

Prospective, single center.

**Population:**

Women with suspicious findings on mammography, tomosynthesis, or ultrasound, and no contraindications for L‐CEM or CE‐MRI. Eighty women were included.

**Field Strength/Sequence:**

1.5 and 3T CE‐MRI, standard protocol for breast, with dedicated coils, according to international guidelines. L‐CEM was performed using a dedicated prototype.

**Assessment:**

Three, off‐site, blinded readers evaluated the images according to the BI‐RADS lexicon in a randomized order, each in two separate reading sessions. Histology served as a gold standard.

**Statistical Test:**

Lesion detection rate, sensitivity, specificity, and negative and positive predictive values (NPV, PPV) were calculated and compared with multivariate statistics.

**Results:**

Included were 80 women (mean age, 54.3 years ±11.2 standard deviation) with 93 lesions (32 benign, 61 malignant). The detection rate was significantly higher with CE‐MRI (92.5–94.6%; L‐CEM 79.6–91.4%, *P* = 0.014). Sensitivity (L‐CEM 65.6–90.2%; CE‐MRI 83.6–93.4%, *P* = 0.086) and NPV (L‐CEM 59.6–71.4%; CE‐MRI 63.0–76.5%, *P* = 0.780) did not differ between the modalities. Specificity (L‐CEM 46.9–96.9%; CE‐MRI 37.5–53.1%, *P* = 0.001) and PPV (L‐CEM 76.4–97.6%; CE‐MRI 73.3–77.3%, *P* = 0.007) were significantly higher with L‐CEM. Variations between readers were significant for sensitivity and NPV. The accuracy of L‐CEM was as good as CE‐MRI (75.3–76.3% vs. 72.0–75.3%, *P* = 0.514).

**Data Conclusion:**

L‐CEM showed a high sensitivity and accuracy in women with suspicious findings on conventional imaging. Compared to CE‐MRI, L‐CEM has the potential to increase specificity and PPV. L‐CEM might help to reduce false‐positive biopsies while obtaining sensitivity comparable to that of CE‐MRI

**Level of Evidence:**

1

**Technical Efficacy Stage:**

2 J. Magn. Reson. Imaging 2020;52:589–595.

## Introduction

Contrast‐enhanced magnetic resonance imaging (CE‐MRI) is the most sensitive technique for breast cancer detection. CE‐MRI has traditionally been used as a second‐line imaging method to solve diagnostic problems in patients with equivocal findings on mammography or ultrasound and staging in patients with a known cancer. Over the past years the use of MRI in a screening setting has also increased.^1^ Some limitations persist, which prevent a wider use of CE‐MRI such as high costs, long acquisition time, and a nonnegligible amount of additional work‐up needed for MRI‐only findings.^2^

Contrast‐enhanced mammography (CEM) is a new imaging modality, technically similar to a digital mammography, which allows the evaluation of lesion enhancement.^3^, ^4^, ^5^, ^6^ Initial studies have shown that CEM is very sensitive for breast cancer detection,^3^, ^7^, ^8^ and it is very well accepted by patients.^9^, ^10^ Up to now, only a few studies have compared CEM to CE‐MRI,^6^, ^11^, ^12^, ^13^, ^14^, ^15^ and most of those studies were retrospective. Nevertheless, the studies indicated that CEM has the potential to become an alternative to CE‐MRI.^6^, ^9^, ^10^, ^11^, ^12^, ^14^, ^16^, ^17^

A drawback of CEM is the use of ionizing radiation. Radiation exposure for CEM is higher than that of full‐field digital mammography and can be higher than that of digital breast tomosynthesis.^18^, ^19^, ^20^ An effort to further reduce radiation exposure with CEM is required. By removing the antiscatter grid, dose reduction can be achieved. A software‐based scatter‐correction method^21^, ^22^ can then be applied to maintain a good image quality.

The aim of the current study was to evaluate the diagnostic performance of a prototype of CEM that allows a low‐dose acquisition (L‐CEM), compared to breast CE‐MRI in women with suspicious findings on conventional imaging.

## Materials and Methods

The institutional Ethics Committee approved this prospective, single‐center study and all regulatory approvals were granted (NCT02608281). All patients included gave their written, informed consent. The study was supported by a grant from Siemens Healthineers (Erlangen, Germany) and Guerbet (Villepinte, France). Authors had full control of all data and statistical results.

### *Patient Population and Standard of Reference*

Eligibility criteria were: women 21 years of age or older, with suspicious findings detected during a screening examination on mammography, tomosynthesis, and/or ultrasound (Breast Imaging Reporting and Data System [BI‐RADS] 4 or 5) at our institution.

Exclusion criteria were: pregnant or lactating women; women who had already undergone surgery for breast cancer; women with breast implants; women undergoing neoadjuvant chemotherapy; women unable to give written, informed consent; women with contraindications to MRI; and women with contraindications to gadolinium‐based and/or iodine‐based contrast media.

All patients who agreed to participate in the study underwent CE‐MRI and bilateral L‐CEM within a minimum interval of 24 and a maximum interval of 72 hours. L‐CEM was performed either before or after CE‐MRI, depending on equipment availability.

In all the included patients, image‐guided biopsy of the most suspicious lesion was performed. Biopsies were performed under ultrasound, stereotactic, or MRI guidance, depending on the visibility of the lesions. All biopsies were performed after both L‐CEM and CE‐MRI were acquired. A marker was positioned in the lesion after each biopsy. Histology was considered as standard of reference. All lesions that presented with a benign aspect on imaging (mammography und ultrasound), and for which a biopsy was not deemed necessary, were excluded.

Data on adverse events due to the contrast agent applications were collected.

### *Low‐Dose CEM Acquisitions and Average Glandular Dose Calculation*

L‐CEM was performed with a modified Siemens Mammomat Inspiration full‐field digital mammography (FFDM) unit (Siemens Healthineers). Both low‐ and high‐energy images were acquired without an antiscatter grid. For breasts with a thickness greater than 70 mm, the antiscatter grid was used. An antiscatter grid is usually placed between the detector and the breast to reduce the amount of Compton‐scattered x‐rays. The grid has a series of lead septa that absorb x‐rays that do not travel parallel to the primary beam. In mammography, even slight amounts of scatter reduce the high contrast required for subtle soft‐tissue imaging. A software‐based scatter correction method^23^ was applied to the images. The Progressive Reconstruction Intelligently Minimizing Exposure (PRIME) algorithm has been approved by the FDA. Scatter kernels were optimized for high‐energy images. Studies have shown that grid‐less image acquisition in mammography and the application of PRIME can reduce the average glandular dose (AGD) up to 27%. 22, 24For L‐CEM, a pair of high‐ and low‐energy images was obtained consecutively during a single breast compression. High‐energy images were obtained with an additional titanium filter and were acquired with a tungsten anode and a 1‐mm titanium filter at a fixed tube voltage of 49 kVp. Low‐energy images were acquired with a tungsten anode target and a 55‐μm rhodium filter at a tube voltage of 28–32 kVp according to regular mammographic imaging protocols in which the photon energies are applied well below the K‐edge of iodine. Exposures were obtained with an automatic image acquisition technique for both energies and standard PRIME acquisition parameters for the low‐energy images. The low‐energy image acquisition and processing parameters were therefore equivalent to standard mammograms.

The total number of mammography views in this study was 640. In only 36 (5.6%, 12 craniocaudal and 24 mediolateral‐oblique views) was an antiscatter grid used, as the breast thickness was greater than 70 mm.^5^, ^6^, ^25^

AGD (mGy) was obtained from the acquisitions monitor for each examination, and compared as a function of breast thickness.^26^

### *Contrast Agent Administration*

Intravenous injection of the contrast agent was performed prior to positioning and breast compression, with the patient in a seated position. A single dose of 2 mL/kg body weight of nonionic iodine contrast agent (Iobitridol/Xenetix 350, Guerbet, Villepinte, France) was administered at a rate of 3 mL/s using a power injector (Ulrich Medical, Ulm, Germany), followed by a saline flush of 20 mL. Sixty to 120 seconds after administration of the contrast agent, the breast was compressed with standard compression force and imaging began, so that the early‐phase enhancement of breast lesions was reached. Breast positioning was equal to that of conventional mammograms. High‐energy images, which primarily carry the iodine information, were performed first to maximize the dual‐energy contrast. Low‐energy images were performed after a delay of 30 seconds.^15^, ^16^

### *CE‐MRI Acquisition*

CE‐MRI of the breast was performed either at our institution (*n* = 48, 60%) or at outside facilities (*n* = 32, 40%). All examinations were performed with either a 1.5T or 3T scanner, with at least eight channel coils and the patients in a prone position. In all cases included, a standard protocol, including a T2‐weighted sequence and a gradient echo T_1_‐weighted sequence before and after the injection of a single dose of a gadolinium‐based contrast agent was performed, in accordance with several guidelines.^1^, ^27^, ^28^ At least three postcontrast series were acquired and subtracted images were available. Details on the sequences acquired are given as Supplemental Material (Table S1).

### *Image Analysis*

Three independent, off‐site readers, with more than 15 years of experience in CE‐MRI and more than 5 years of experience in CEM, evaluated all images in two separate reading sessions. Each reader was blinded to all clinical and radiological information.

Readings were performed on a dedicated workstation (syngo.Breast Care; Siemens Healthcare) with high‐resolution monitors (8MP Monitor, 12 BIT, Monitor‐Pixel: 0,17 mm × 0,17 mm; Brightness/Luminance: >2100 cd/m^2^). Before data collection, all readers analyzed a series of 20 test cases with L‐CEM to become familiar with the typical image appearance of the device used. The test cases were not part of the final reading. All cases were displayed in each session: half with CE‐MRI and half with L‐CEM. Each case was presented only once per reading session. Reading sessions were separated by a washout period of at least 4 weeks to avoid memory bias.

The readers were asked to define:

- The presence/absence of a lesion, lesion location (breast quadrants), and lesion type (for L‐CEM: mass, microcalcifications, asymmetry, distortion; for CE‐MRI: mass, nonmass enhancement);
- Lesion size (mm);
- BI‐RADS^29^ score for each lesion.

The same BI‐RADS descriptors used for the evaluation of CE‐MRI were also applied for L‐CEM.^6^ Only one lesion per breast was considered. When more lesions were described, only the most suspicious lesion per breast, for which a histological verification was available, was considered.

### *Statistical Analysis*

All statistical computations were performed using IBM SPSS Statistics for Windows v. 24.0.2 (Armonk, NY). Metric data are described using means and standard deviation (SD). Nominal data are presented using absolute frequencies and percentages. The analysis was performed on a per‐breast basis. If more than one lesion was present, only the most suspicious lesion was considered for the analysis. The detection rate was calculated considering the number of lesions detected by the reader on the absolute number of lesions biopsied and included in the analysis (benign and malignant). In addition, the detection rate was calculated separately for benign and malignant lesions. The detection rate for benign lesions was calculated considering the number of benign lesions identified by the reader on the total number of benign lesions included in the analysis, regardless of the BI‐RADS classification. The same method was used to calculated the detection rate for malignant lesions.

Examinations classified as BI‐RADS 1–3 were considered negative (nonsuspicious), and BI‐RADS 4 and 5 were considered positive (suspicious). For sensitivity, detected lesions classified as nonsuspicious and undetected lesions were treated as false‐negatives. Specificity was based on all breasts. Positive and negative predictive values (PPV, NPV) were also assessed.

Generalized estimating equations (GEE) were used to compare detection rates, sensitivity, specificity, accuracy, and NPV and PPV for the two examinations and the three readers. GEE was used to take into account the effect of multiple readers and multiple reading methods for the multiple lesions and repeated measures per lesion. A *P*‐value equal to or below 0.05 was considered to indicate significant results.

A sample size of 80 was selected to provide a power of 80% for a difference in accuracy of 10% when comparing L‐CEM to CE‐MRI, and assuming 11% discordant ratings.

## Results

### *Lesion Characteristics*

**Figure 1:**
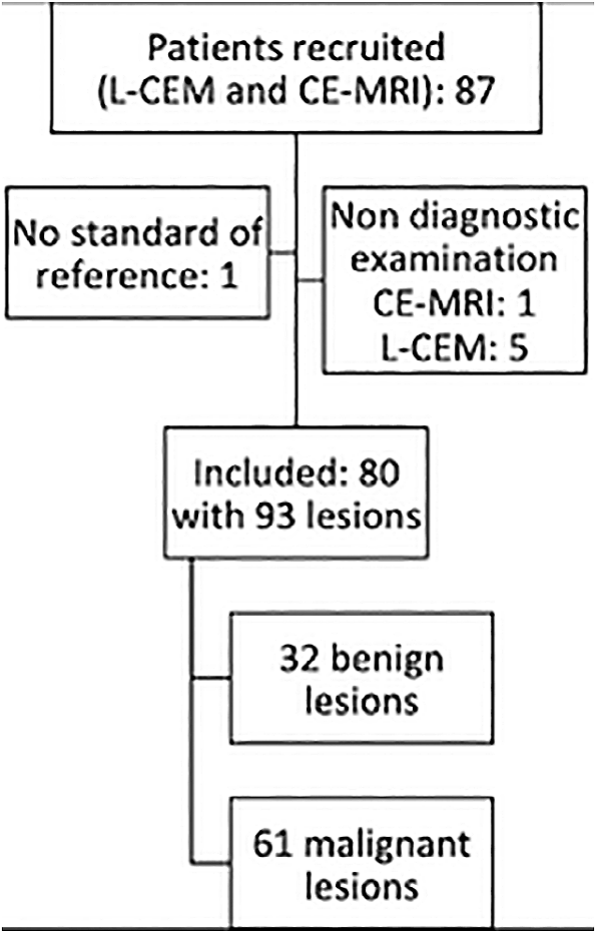
Flow chart with the patients and lesions included in the study. L‐CEM: low‐dose, contrast‐enhanced mammography; CE‐MRI: contrast‐enhanced magnetic resonance imaging.

A total of 80 patients (mean age, 54.3 years; range, 34–83 years; SD 11.2) with 93 histologically verified breast lesions were included in the analysis (Fig. 1). There were 32 benign and 61 malignant breast lesions (Table 1) seen in 93 breasts. Sixty‐seven breasts presented with no lesions.

**Table 1:** Histology of the 93 Histologically Verified Lesions Included in the Analysis

	Number (%)
**Malignant**	**61**
Invasive carcinoma with ductal carcinoma in situ	25 (41)
Invasive carcinoma NST*	19 (31)
Ductal carcinoma in situ	15 (25)
Invasive lobular carcinoma	2 (3)
**Benign**	**32**
Fibrocystic changes	15 (47)
Papilloma	6 (19)
Inflammatory changes	6 (19)
Fibroadenoma	5 (15)

NST: nonspecial type.

All patients tolerated the contrast agent application and no adverse reactions were noted.

Lesion size ranged from 4–120 mm for L‐CEM (mean and standard deviation for reader 1, 22.6 ± 20.7 mm; for reader 2, 23.9 ± 20.7 mm; for reader 3, 23.3 ± 21.7 mm) and from 4–100 mm with CE‐MRI (mean and standard deviation for reader 1, 21.9 ± 15.3 mm; for reader 2, 25.1 ± 17.8 mm; for reader 3, 23.5 ± 15.6 mm). Lesions presented on L‐CEM as a mass enhancement in 66 cases (71.0%) and as a nonmass enhancement in 19 cases (20.4%). The remaining eight (8.6%) lesions were microcalcifications on mammography, without associated enhancement. Lesions presented on CE‐MRI as a mass in 75 cases (80.7%) and as a nonmass in 18 (19.3%).

### *Diagnostic Performance*

Results for lesion detection rate, sensitivity, specificity, and NPV, PPV of L‐CEM and CE‐MRI are summarized in Tables 2 and 3.

**Table 2:** Detection Rate (Number of Lesion Detected on the 93 Lesions Included in the Analysis), With Confidence Intervals, of Low‐Dose, Contrast‐Enhanced, Dual‐Energy Mammography (L‐CEM) and Contrast‐Enhanced Magnetic Resonance Imaging (CE‐MRI) for the Three Readers

	Reader 1 (%)	Reader 2 (%)	Reader 3 (%)
Overall			
L‐CEM	79.6	83.9	91.4
	70.7–86.3	74.6–90.2	84.1–95.5
CE‐MRI	93.6	94.6	92.5
	86.5–97.0	87.9–97.7	85.0–96.4
Benign lesions			
L‐CEM	59.4	71.9	84.4
	42.2–74.5	54.5–84.6	67.8–93.6
CE‐MRI	93.7	93.7	84.4
	78.8–99.3	78.8–99.3	67.8–93.6
Malignant lesions			
L‐CEM	90.2	90.2	95.1
	79.8–95.7	79.8–95.7	86.0–98.9
CE‐MRI	93.4	95.1	96.7
	83.9–97.9	86.0–98.9	88.1–99.7

**Table 3:** Sensitivity, Specificity, Diagnostic Accuracy, Negative and Positive Predictive Values (NPV, PPV) for the Three Readers With Low‐Dose, Contrast‐Enhanced, Dual‐Energy Mammography (L‐CEM) and Contrast‐Enhanced Magnetic Resonance Imaging (MRI)

	Reader 1 (%, 95% CI)	Reader 2 (%, 95% CI)	Reader 3 (%, 95% CI)
Sensitivity			
L‐CEM	65.6	86.9	90.2
	53.5–75.9	76.7–93.0	80.6–95.3
MRI	83.6	90.2	93.4
	72.4–90.8	79.9–95.5	83.9–97.5
Specificity			
L‐CEM	96.9	56.3	46.9
	80.8–99.6	38.5–72.6	31.3–63.3
MRI	53.1	37.5	40.6
	36.7–68.9	23.2–54.3	25.8–57.3
Accuracy			
L‐CEM	76.3	76.3	75.3
	67.0–84.0	67.0–84.0	66.0–83.0
MRI	73.1	72.0	75.3
	63.0–81.0	62.0–80‐0	66.0–83.0
NPV			
L‐CEM	59.6	69.2	71.4
	45.2–72.5	49.1–84.0	49.2–86.6
MRI	63.0	66.7	76.5
	43.4–79.1	42.9–84.2	51.4–90.9
PPV			
L‐CEM	97.6	79.1	76.4
	84.6–99.7	67.1–87.6	64.6–85.2
MRI	77.3	73.3	75.0
	65.4–86.0	61.5–82.6	63.5–83.8

The overall detection rate was significantly higher with CE‐MRI compared to L‐CEM (*P* = 0.014). When considering benign and malignant lesions separately, a significantly higher detection rate with CE‐MRI was seen for benign, but not for malignant lesions.

Sensitivity and NPV were high for both L‐CEM and CE‐MRI (Table 3). Sensitivity (*P* = 0.086) and NPV (*P* = 0.78) did not differ among the techniques used.

L‐CEM showed a significantly higher specificity (*P* = 0.001) and a higher PPV (*P* = 0.007) than CE‐MRI. The accuracy of L‐CEM was as high as that of CE‐MRI (*P* = 0.514).

GEE showed that sensitivity, NPV, and accuracy were not dependent on the modality used (*P* = 0.086, *P* = 0.78, and *P* = 0.514, respectively).

Both sensitivity and NPV varied between readers. GEE showed that both sensitivity and NPV were dependent on the reader (*P* < 0.001 and *P* = 0.032). Accuracy was not dependent on the readers (*P* = 0.913).

Variability, with regard to specificity and PPV, was dependent on both modality used (*P* = 0.001 and *P* = 0.007) and reader (*P* = 0.001 and *P* = 0.032) (Table 3).

### *False‐Positives and False‐Negatives*

Lesions classified as false‐negatives by all three readers were two with CE‐MRI (two ductal carcinomas in situ) and four with L‐CEM (two invasive ductal carcinomas and two ductal carcinomas in situ).

**Figure 2:**
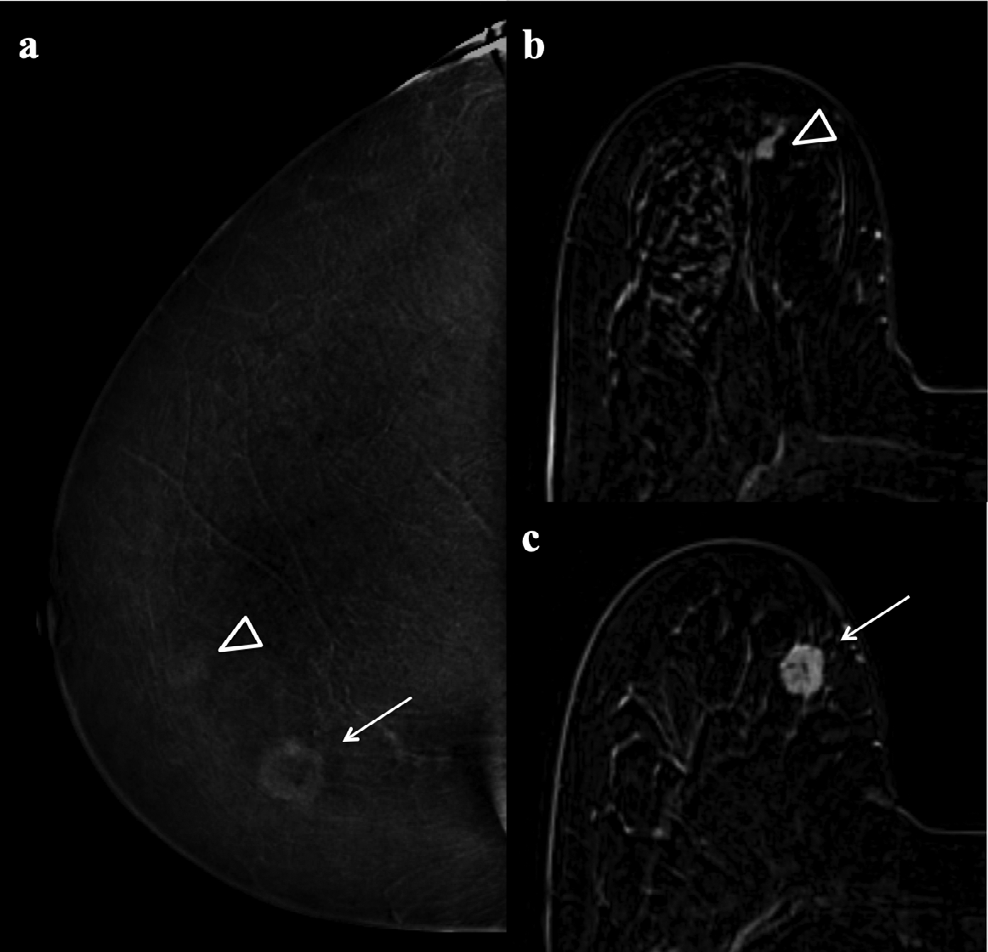
Low‐dose contrast‐enhanced mammography (L‐CEM, **a**) and contrast‐enhanced MRI of the breast (second subtracted image, **b** and **c**) in a 69‐year‐old woman. Histology revealed an invasive ductal carcinoma in the right breast (arrow in a and c). The lesion shows heterogeneous enhancement and irregular margins in both L‐CEM and MRI and was considered suspicious. A second enhancing lesion with indistinct margins was clearly visible on MRI (arrowhead in b) and considered suspicious as well. In contrast, the same lesions shows only a minimal, nonsuspicious enhancement on L‐CEM (arrowhead in a). Histology showed a benign lesion (papilloma without atypia).

Lesions classified as false‐positives by all three readers were 13 with MRI (three fibroadenomatoid hyperplasias, two fibrocystic changes, two papillomas, two fat necroses, two inflammatory changes, one PASH, one fibroadenoma) and two with L‐CEM (one atypical ductal hyperplasia and one fat necrosis) (Fig. 2).

### *Average Glandular Dose*

**Figure 3:**
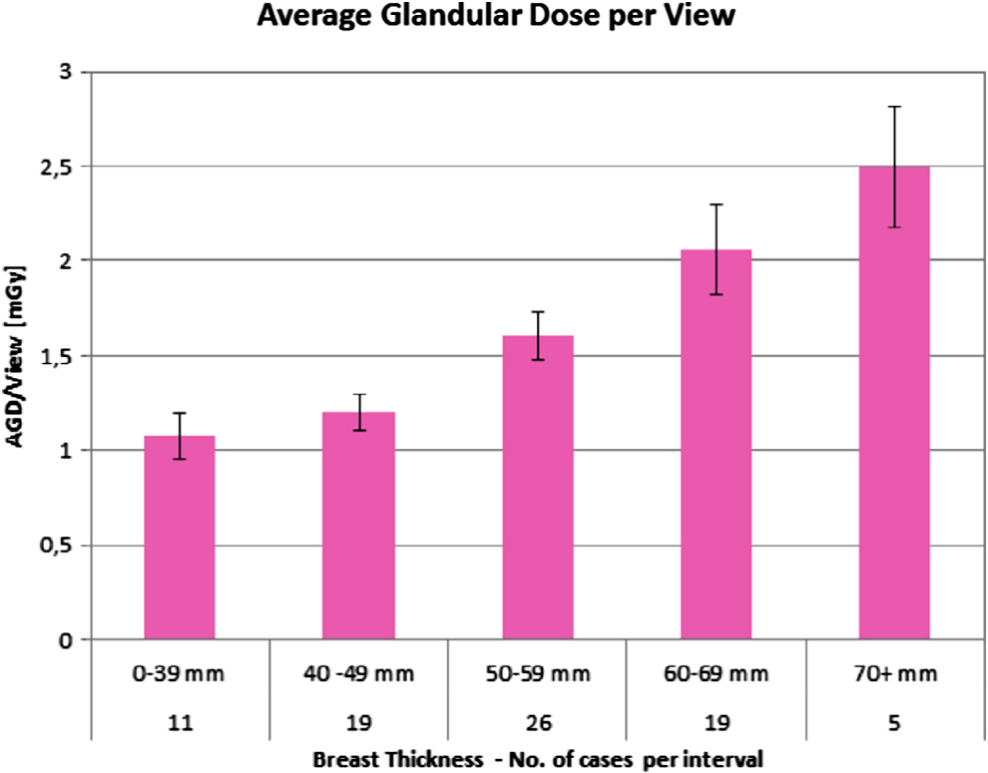
Mean average glandular dose (AGD) and standard deviation calculated per view for increasing breast thickness intervals. As expected, AGD increased with increased breast thickness. Despite this, the low‐dose, contrast‐enhanced mammography system used was able to keep the AGD below 2.49 mGy.

The AGD distribution for L‐CEM per view is shown in Fig. 3 and ranged, according to breast thickness, from 1.07–2.49 mGy.

## Discussion

Our results indicate that L‐CEM offers high sensitivity and accuracy in the assessment of breast findings classified as suspicious on conventional imaging. Compared to CE‐MRI, L‐CEM has the potential to increase specificity and PPV. L‐CEM reduces unnecessary biopsies for benign lesions while obtaining sensitivity comparable to that of CE‐MRI.

Our results confirm a high sensitivity for L‐CEM in breast cancer detection. All three readers of our study had comparable sensitivities using L‐CEM and CE‐MRI. The per‐lesion sensitivity found in a multireader study on patients with breast cancer ranged between 72% and 80% for CE‐MRI and from 66% to 77% for CEM.^11^ The results were similar to those obtained in our analysis. A high sensitivity, with no significant differences between CEM and CE‐MRI, was found by several studies.^12^, ^13^, ^14^, ^30^ It must be underlined that in all these studies CE‐MRI consistently showed a slightly higher sensitivity, despite the absence of a statistically significant difference between the two methods. Larger studies are needed to confirm if CEM could indeed be a safe alternative to breast CE‐MRI in all patients.

The specificity of L‐CEM was higher than that of CE‐MRI. The specificity of CEM is currently a topic of discussion, as early studies have shown highly variable results.^3^ The variability in results was most likely related to the case selection in a defined study setting and, in part, to the developing initial technology.^31^, ^32^ Recent studies, characterized by the application of more advanced technologies, improved image quality, increased reader experience, and a larger patient population, showed that CEM is characterized by a high specificity, approaching 90% and a PPV higher than CE‐MRI.^11^, ^13^, ^15^ Similar results were seen in our study where the detection rate of benign lesions (false‐positives) with L‐CEM was lower than that with CE‐MRI. It seems that many benign lesions were considered suspicious due to the evident enhancement on CE‐MRI, whereas they were assessed accurately with L‐CEM due to the absence of enhancement.

False‐negative results were obtained with both CE‐MRI and L‐CEM. CE‐MRI missed two ductal carcinomas in situ. It is already known that, despite the high sensitivity, CE‐MRI can overlook some intraductal lesions, due to the absence of detectable enhancement.^33^, ^34^ These lesions can usually be seen on mammography and, thus, on CEM, due to the presence of microcalcifications. This was the case for the two CE‐MRI false‐negatives in our study. On L‐CEM, an invasive ductal carcinoma was overseen, which was detected on CE‐MRI. Similar results of missed IDCs were found by Lalji et al^35^ and Thibault et al^17^ Lesion size, lesion location, presentation at mammography as an asymmetry rather than a mass, and marked background enhancement might be some of the possible causes of false‐negative results with CEM.

Based on our results, L‐CEM might help to reduce false‐positive biopsies while increasing the cancer detection rate. This is of great advantage, as CE‐MRI of the breast is a rather expensive examination. In addition, due to its high lesion detection and the challenge of characterizing small and nonmass enhancements, CE‐MRI often leads to biopsies for lesions that prove to be benign. L‐CEM could be a cost‐effective alternative^36^ and might help to improve the management of patients with suspicious or inconclusive findings on mammography, tomosynthesis, or ultrasound.

An effort to further reduce radiation exposure with CEM is required, as AGD is significantly higher for CEM, compared to digital mammography or even tomosynthesis.^19^, ^20^ A significant reduction in radiation dose for mammography has been obtained by improving tubes and by optimizing detector materials and structures. Another method to reduce radiation dose is to remove the antiscatter grid. In our study, we used an L‐CEM prototype without an antiscatter grid but with dedicated, software‐based scatter‐correction method. By using our scatter‐correction algorithm, the AGD for breasts with a thickness of 55 mm was 1.6 mGy. In contrast, CEM systems with an antiscatter grid showed an AGD between 2.2 and 2.4 mGy.^19^, ^20^ Overall, our system allows a reduction in AGD between 21% and 48%, depending on breast thickness.

## Limitations

One limitation of this study is that the MRI scans were acquired with different field strengths and sequence parameters. Both 1.5T and 3T scanners are considered adequate for CE‐MRI of the breast.^27^ A recent study showed no significant difference in the diagnostic performance of 1.5T or 3T scanners.^37^ In addition, all MRI examinations were performed using international guidelines and recommendations.^27^, ^28^ Thus, this limitation should be considered minor and is unlikely to have influenced the results of the CE‐MRI readings. Even more, we could still prove an excellent performance of our readers and MRI results, which are comparable to other studies comparing CEM with CE‐MRI.^6^, ^11^, ^14^, ^38^

We did not include cases for which a histological evaluation was not available; thus, our study is associated with a high malignancy rate, as well as the minor risk of missing a single, false‐negative finding that might have been detected if follow‐up were available. The analysis was performed on a per‐breast basis; thus, the accuracy of L‐CEM vs. CE‐MRI to analyze multifocality or multicentricity was not assessed.

## Conclusion

L‐CEM showed a high detection rate for malignant lesions. The sensitivity and accuracy of L‐CEM were as good as that of CE‐MRI. In addition, L‐CEM showed a higher specificity and PPV. Based on our results, L‐ CEM might help to reduce false‐positive biopsies while obtaining a sensitivity comparable to that of CE‐MRI.

## Supporting information


**Table S1.** Vendors and sequences of the MRI examinations included in the study. All images were acquired in the axial plane.
