# Supplementary material for: Low‐Dose, Contrast‐Enhanced Mammography Compared to Contrast‐Enhanced Breast MRI: A Feasibility Study
Source: J Magn Reson Imaging. 2020 Feb 14;52(2):589–95. doi: 10.1002/jmri.27079 (PMC7496227; doi:10.1002/jmri.27079)
Supplement: Supplementary file 1 — Table S1. Vendors and sequences of the MRI examinations included in the study. All images were acquired in the axial plane. [file JMRI-52-589-s001.docx]

**Table A**

Vendors and sequences of the MRI examinations included in the study. All images were acquired in the axial plane.

| **Vendor** | Siemens | Siemens | Siemens | Philips |
| --- | --- | --- | --- | --- |
| **Tesla** | 3 | 1.5 | 1.5 | 1.5 |
| **T1- weighted sequences** | | | | |
| **Sequence** | TWIST* | fl3D Dixon** | fl3D dynamic*** | T1 FFE3D ° |
| **TR** | 6.23 | 10 | 8.9 | 7.05 |
| **TE** | 2.95 | 2.39 | 4.7 | 4.6 |
| **Matrix** | 384x384 | 521x410 | 448x403 | 340x340 |
| **Slices** | 144 | 80 | 40 | 132 |
| **Temporal resolution** | 28s, one pre- and 10 post-contrast | 70s, one pre- and 3 post-contrast | 110s, one pre- and 5 post-contrast | 72s, one pre- and 3 post-contrast |
| **T2- weighted sequences** | | | | |
| **Sequence** | T2-TSE | T2-TSE | TIRM Blade | eSTIR |
| **TR** | 4630 | 3990 | 6990 | 4039.91 |
| **TE** | 194 | 183 | 121 | 65 |
| **TI** | / | / | 160 | 175 |
| **Matrix** | 640x480 | 512x384 | 320x320 | 263x223 |
| **Slices** | 65 | 48 | 32 | 50 |

*View-sharing, 3D, time-resolved angiography with stochastic trajectory, gradient echo sequence

** 3D fast low‐angle shot *T*_1_ Dixon sequence

*** 3D fast low‐angle shot anisotropic *T*_1_‐weighted sequence without fat saturation

° Gradient echo 3D without fat suppression
